# Intention to Receive the COVID-19 Vaccination in China: Application of the Diffusion of Innovations Theory and the Moderating Role of Openness to Experience

**DOI:** 10.3390/vaccines9020129

**Published:** 2021-02-05

**Authors:** Phoenix Kit-han Mo, Sitong Luo, Suhua Wang, Junfeng Zhao, Guohua Zhang, Lijuan Li, Liping Li, Luyao Xie, Joseph T. F. Lau

**Affiliations:** 1 Centre for Health Behaviours Research, JC School of Public Health and Primary Care, The Chinese University of Hong Kong, Hong Kong, China; phoenix.mo@cuhk.edu.hk (P.K.-h.M.); sitongluo@cuhk.edu.hk (S.L.); 1155149225@link.cuhk.edu.hk (L.X.); 2 Graduate School of Baotou Medical College, Baotou Medical College, Baotou 014040, China; 101991063@btmc.edu.cn; 3 Department of Psychology, School of Education, Henan University, Kaifeng 475100, China; zhaojf@henu.edu.cn; 4 Department of Psychology, School of Psychiatry, Wenzhou Medical University, Wenzhou 325035, China; zghcnu@wmu.edu.cn; 5 School of Public Health, Dali University, Dali 671003, China; lelejuan@eastern-himalaya.cn; 6 Medical College, Shantou University, Shantou 515041, China; lpli@stu.edu.cn

**Keywords:** COVID-19 vaccination, diffusion of innovations theory, perceived efficacy of vaccine, social media, openness to experience, descriptive norm

## Abstract

COVID-19 has caused a devastating impact on public health and made the development of the COVID-19 vaccination a top priority. Herd immunity through vaccination requires a sufficient number of the population to be vaccinated. Research on factors that promote intention to receive the COVID-19 vaccination is warranted. Based on Diffusion of Innovations Theory, this study examines the association between the perceived efficacy of the COVID-19 vaccination, use of social media for COVID-19 vaccine-related information, openness to experience and descriptive norm with the intention to receive the COVID-19 vaccination, and the moderating role of openness to experience among 6922 university students in mainland China. The intention to receive the free and self-paid COVID-19 vaccination is 78.9% and 60.2%, respectively. Results from path analyses show that perceived efficacy of the COVID-19 vaccination, use of social media for COVID-19 vaccine-related information, and openness to experience and descriptive norm are all positively associated with the intention to receive COVID-19 free and self-paid vaccination. The association between the perceived efficacy of the COVID-19 vaccination and descriptive norm with the intention to receive the COVID-19 vaccination is stronger among those with a lower level of openness to experience. Our findings support the usefulness of Diffusion of Innovations Theory and the moderating role of openness of experience in explaining intention to receive the COVID-19 vaccination.

## 1. Introduction

The emergence of coronavirus SARS-CoV-2 (severe acute respiratory syndrome coronavirus 2, the COVID-19) strain and its devastating impacts on global health and economy have made it a top priority to develop effective and safe vaccines for this disease [1]. As of 8 December 2020, over 65.8 million cases and 1.5 million deaths were reported cumulatively over the globe [2]. There is no doubt that vaccination will be one of the most effective strategies to prevent the further spread of the disease. Since the COVID-19 genetic sequence was published in January 2020, more than 100 COVID-19 vaccine candidates have been rushing into development [3], some of which are already in clinical trials.

The public availability of vaccination is crucial, since herd immunity can be achieved through large-scale vaccination programs [4], thereby protecting the lives of the most vulnerable groups and reducing the social and economic burden of the current crisis. According to a recent study, the predicted vaccine coverage of 55% to 82% of the population is needed to achieve COVID-19 herd immunity [5]. Herd immunity through vaccination requires a sufficient proportion of the population to be vaccinated, and its effectiveness also depends on the individual’s willingness to be vaccinated. A report showed that 73.9% of individuals in seven European countries intended to get vaccinated against COVID-19 [6].

### 1.1. Factors Associated with the Intention to Receive the COVID-19 Vaccination

The reasons behind the decision to receive vaccinations varied. Studies investigating factors associated with uptake of vaccinations, such as influenza vaccinations, have demonstrated that vaccination uptake was influenced by a range of personal and interpersonal factors [7,8]. Current studies on factors related to willingness to vaccinate against COVID-19 have also identified a number of demographics, cognitive and psychosocial factors of intention to receive the COVID-19 vaccination, including age, gender, education, having insurance, attitudes toward the vaccine, confidence in government information, perceived susceptibility to COVID-19, and perceived benefits and side effects of the vaccine [6,9,10,11,12,13,14,15,16,17]. Some behavioral theories, such as the Theory of Planned Behavior and the Health Belief Model, have also been used to explain COVID-19 vaccination intention [13,18].

### 1.2. Application of the Diffusion of Innovations (DOI) Theory on Intention to Receive the COVID-19 Vaccination

It is no doubt that the COVID-19 vaccination is an innovative behavior; a framework that can explain the uptake of the novel vaccine is, therefore, essential. Most of the behavioral theories used to explain COVID-19 vaccination acceptance so far failed to explain how an innovative behavior will be adopted in the early phase and gradually become diffused as a common practice. The DOI is one of the most popular theories for understanding the adoption of innovative behavior [19]. The theory proposes four factors that would explain the uptake of innovative behavior: (1) Attributes of the innovation; (2) communication channel; (3) characteristics of the adopters; and (4) social system. The DOI has been applied to a number of innovative health behaviors, such as the adoption of information technologies in healthcare [20,21], design of diabetes management program [22], and use of HIV Pre-exposure Prophylaxis [23]. It is conjectured that the four factors of the DOI will be useful in explaining an individual’s willingness to receive the novel COVID-19 vaccination.

#### 1.2.1. Attributes of the Innovation—Perceived Efficacy of the COVID-19 Vaccination

Attributes of the innovation refer to several perceived qualities, such as relative advantage, compatibility, complexity, trialability, and observability of the innovation. Among which, the relative advantage is the most relevant construct to explain the intention to adopt the COVID-19 vaccination. In the COVID-19 pandemic, the key concerns about the COVID-19 vaccination were mainly about safety, efficacy, and need for new vaccines [24,25]. High perceived benefits and perceived efficacy of the vaccine were associated with greater COVID-19 vaccine acceptance [6,9,10,11,12,13,14], while fear of side effects and negative views on vaccine efficacy and safety have been suggested as factors for COVID-19 vaccine hesitancy [15,16,17].

#### 1.2.2. Communication Channels—Social Media Use for COVID-19 Vaccine-Related Information

Communication channels refer to the medium through which people obtain information about the innovation and perceive its usefulness. In recent decades, social media has been increasingly used as a platform for searching health information [26]. During the COVID-19 outbreak, social media has functioned as a substitute for traditional resources for rapid epidemic information sharing and gathering, thereby avoiding the risk of infection caused by social contact [27]. Using social media for COVID-19 related information could increase individuals’ knowledge about the disease and inform them about health care decisions, thereby promoting their health behaviors. On the other hand, social media has also been identified as a major channel for the dissemination of conspiracy beliefs and misinformation [28]. Studies have found that the use of social media as a source of information about COVID-19 was positively associated with COVID-19 conspiracy beliefs and negatively associated with COVID-19 health-protective behaviors [29]. There is also an increasing concern that the use of social media for COVID-19 vaccine-related information may promote vaccine hesitancy, thereby decreasing the intention to receive the COVID-19 vaccination [30].

#### 1.2.3. Characteristics of the Adopter—Openness to Experience

Characteristics of the adopter refer to the level of innovativeness and openness of the individual. According to the DOI, innovators and early adopters of innovation have been repeatedly shown to be more adventurous, open to change, and attracted to new ideas and practices [19]. In particular, openness to experience (OE) is a fundamental dimension of personality, including intellectual curiosity, aesthetic sensitivity, liberal values, and emotional differentiation [31]. OE distinguishes those who prefer and seek out novelty and variety from those who prefer and seek out familiarity and routine. People with a high degree of OE tend to have behavioral and mental flexibility, while people low in openness are generally characterized as being more closed-minded, less curious, and preferring familiar people, places, and things [32,33]. Previous research consistently showed OE was positively related to creativity and innovation, and people with high OE were more likely to engage in innovative behaviors [34,35,36]. OE was also found to be associated with health-promoting behaviors and preventive healthcare utilization, including influenza vaccination [37,38,39]. From our understanding, no studies have examined the role of OE on the intention to receive COVID-19 intention.

#### 1.2.4. Social System—Descriptive Norm

The social system refers to the structure of the social system that might affect the individual’s attitudes towards innovation. The perception of which the behavior will be performed by the majority of the population within the social system, known as the descriptive norm, has been found to be an important factor that explains the uptake of innovation [40]. The social pressure created by a descriptive norm may increase one’s intentions to adopt and maintain the innovation. Social norm has been found to be associated with various health behaviors, such as influenza vaccination [41] and HPV vaccination [42]. In the context of COVID-19, greater perceived social norms about vaccination were found to be associated with COVID-19 vaccination intention [18].

### 1.3. The Moderating Role of OE

In DOI, the inclination of adopting an innovative behavior is considered as a direct factor, as well as a moderator, which shapes the attitude, and the norms that impact behavior [19]. It is claimed that for innovators and early adopters, their decision to adopt an innovation is predominately driven by their inherent innovativeness, so relatively little is needed to persuade them to adopt the behavior. On the other hand, those who are less open to adopting an innovation are more likely to be influenced by other attributes and social pressure [43]. Furthermore, people with a high level of OE tend to exhibit nonconforming behavior [33], which may lead them to reject alleged objective knowledge from conventional authority sources and be in favor of a personal, emotional, and spiritual approach to health decisions [44]. Therefore, it is conjectured that the association between the perceived efficacy of the COVID-19 vaccination, use of social media for COVID-19 vaccine-related information, and descriptive norm with the intention to receive the COVID-19 vaccination will be stronger among individuals with a lower level of OE. Such question has not been examined in the extant literature.

### 1.4. The Present Study

Based on the DOI, the present study examined the association between the perceived efficacy of the COVID-19 vaccination, use of social media for COVID-19 vaccine-related information, OE, and descriptive norm with the intention to receive the COVID-19 vaccination among Chinese university students. The moderating role of OE on the association between the perceived efficacy of the COVID-19 vaccination, use of social media for COVID-19 vaccine-related information, and descriptive norm with the intention to receive the COVID-19 vaccination was also examined.

## 2. Methods

### 2.1. Study Design

A cross-sectional online survey was conducted during 1–28 November 2020 among university students in China.

### 2.2. Participants

Target participants were university students. Inclusion criteria were: (a) Full-time student of the selected universities, and (b) able to read and write Chinese.

### 2.3. Procedure

**Figure 1:**
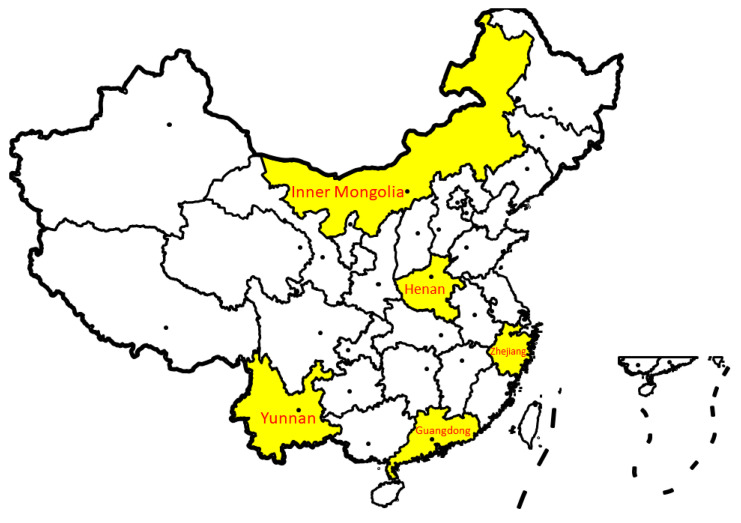
The geographical location of the provinces involved in the present study.

Participants were recruited from five universities from five provinces (Zhejiang, Yunnan, Guangdong, Inner Mongolia, Henan) of mainland China. The five provinces were geographically and socioeconomically representative of mainland China to some extent (see Figure 1 for the geographical location of the five provinces). A total of 165 classes within faculties of arts, sciences, social sciences, economics or management, engineering, and medicine or pharmacy were selected from various grades from the five universities. The teachers or student representatives of the selected classes sent an invitation message with a QR code for the online questionnaire to all the students in the classes via the WeChat groups used for class administration. Participants were informed that the study was voluntary and confidential, and the return of the completed questionnaire implied informed consent.

Participants self-administered the online questionnaire, which took about 10–15 min to complete. Upon completion, the participant could join a lottery which offered eight prizes of 10–50 RMB (about 1.5–7.5 USD) and “lucky money” of 1 RMB to around half of the participants for each university. Ethical approval was obtained from the ethics committee of the corresponding author’s affiliated university. A total of 6940 completed responses were collected (response rate = 72.3%). After excluding 18 participants who have already received the COVID-19 vaccination by the time of the survey, the final sample size was 6922.

### 2.4. Measures

Perceived efficacy of the COVID-19 vaccination was measured by a single item: To what extent do you think the COVID-19 vaccine will be effective in preventing COVID-19 infection? Participants were asked to input a number that represented the percentage of the efficacy of the vaccination.

Social media use for COVID-19 vaccine-related information was measured by a single item: “In the past month, how long have you been exposed to information about the COVID-19 vaccine (e.g., progress of research development, efficacy, safety, price, time for official launch) through social media on an average week?” Items are rated on a 5-point Scale: 0 = almost none, 2 = less than 30 min, 3 = 30 min to 1 h, 4 = 1 to 2 h, 5 = 2 h or more.

Descriptive norm was measured by a single item: “In the first 6 months since the official launch of COVID-19 vaccine in China, how many percentage of the people whom you closely contact with (e.g., classmates/roommates/teachers) will be vaccinated?” Participants were asked to input a number that presented the percentage of the people who will be vaccinated.

OE was measured by the OE subscale of the Ten-Item Personality Inventory (TIPI), a valid brief measure of the Big Five personality with satisfactory psychometric properties [45]. Items were rated on a 5-point Scale from 1 = strongly disagree to 5 = strongly agree. The Cronbach’s alpha of the subscale was 0.72.

Intention to receive the COVID-19 vaccination. Participants were asked to rate their likelihood of receiving the COVID-19 vaccination under the following conditions if the vaccine was launched in China: (1) The vaccine was recommended by the government and offered free; (2) the vaccine was recommended by the government and was self-paid. The items were rated on a 5-point Scale from 1 = definitely no to 5 = definitely yes.

Background characteristics, such as age, gender, ethnicity, discipline of study, and year of study, were recruited.

### 2.5. Data Analysis

Descriptive statistics and correlation between variables were first presented. There are two dependent variables in the present study: Intention to receive the COVID-19 vaccination (free) and intention to receive the COVID-19 vaccination (self-paid). The association between the perceived efficacy of the COVID-19 vaccination, use of social media for COVID-19 vaccine-related information, OE, and descriptive norm with the two dependent variables were tested using path analysis. To test the moderating role of OE on the association between the other three independent variables and the two outcomes, OE and the other three independent variables were first standardized, and interaction terms between OE and each of the three independent variables were created. A series of path analyses were conducted, with each of the independent variables, OE, and their respective interaction terms and the two dependent variables being entered in the model. All background variables there were significant at the *p* < 0.05 level were also adjusted for in the path analyses. The following indexes were used to evaluate the model fit: Comparative fit index (CFI), Incremental Fit Index (IFI), and Root Mean Square Error of Approximation (RMSEA). Analyses were conducted using AMOS 26.0.

## 3. Results

### 3.1. Descriptive Statistics

Around two-thirds of the sample (63.6%) were female, and their mean age was 19.4 years (SD = 1.51). The majority of them were Han ethnicity. About half of them (50.9%) studied in medicine-related discipline, and 43.2% of them were freshmen. The intention to receive the free and self-paid COVID-19 vaccination was 78.9% and 60.2%, respectively (Table 1).

### 3.2. Correlation between Variables

Table 2 shows the correlation between the variables in the study. Among the background variables, gender (reference group: male) was positively correlated with the intention to receive the COVID-19 vaccination (free) (*r* = 0.03, *p*< 0.05) and intention to receive the COVID-19 vaccination (self-paid) (*r* = 0.03, *p*< 0.01). Ethnicity (reference group: Han) had a negative correlation (*r* = −0.05, *p*< 0.001), while discipline of study (reference group: Medicine) had a positive correlation (*r* = 0.04, *p*< 0.01) with the intention to receive vaccination (free). Age (*r* = −0.02, *p*< 0.001) and year of study (*r* = −0.05, *p*< 0.001) had a negative correlation with the intention to receive vaccination (self-paid).

Among the DOI variables, perceived efficacy of the COVID-19 vaccination, social media use for COVID-19 vaccine-related information, OE, and descriptive norm all had a significant positive correlation with the intention to receive the COVID-19 vaccination (free) (*r* = 0.05 to 0.23, *p* < 0.001) and intention to receive the COVID-19 vaccination (self-paid) (*r* = 0.09 to 0.28, *p* < 0.001).

### 3.3. DOI Factors on Intention to Receive the COVID-19 Vaccination

**Figure 2:**
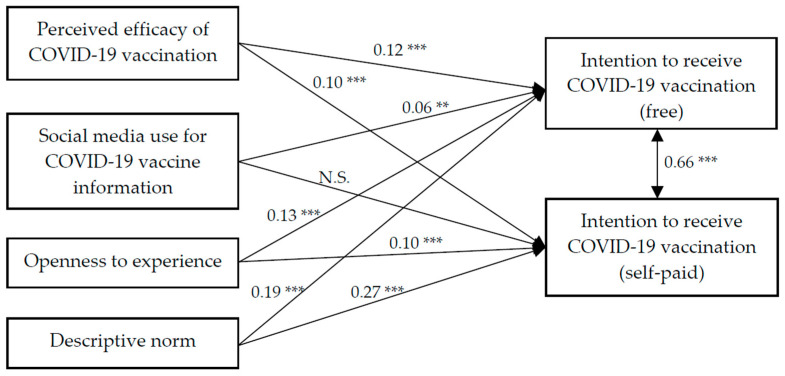
Path analysis of perceived efficacy of the COVID-19 vaccination, social media use, openness to experience, descriptive norm, and intention to receive the COVID-19 vaccination (free and self-paid). ** *p* < 0.01, *** *p* < 0.001, N.S. = not significant. The association between background variables and intention to receive the COVID-19 vaccination (free and self-paid) were omitted for clarity.

Findings of the path analysis showed that the model had a satisfactory fit, χ^2^ (33) = 455.25, CFI = 0.97, IFI = 0.97, RMSEA = 0.03. Perceived efficacy of the COVID-19 vaccination, OE, and descriptive norm had positive association with the intention to receive the COVID-19 vaccination (free) (β = 0.12, 0.13, 0.19, *p* < 0.001, respectively) and intention to receive the COVID-19 vaccination (self-paid) (β = 0.10, 0.10, 0.27, *p* < 0.001, respectively). Social media use for COVID-19 vaccine information had significant association with the intention to receive the COVID-19 vaccination (free) (β = 0.06, *p* < 0.01), but no association with the intention to receive the COVID-19 vaccination (self-paid) (Figure 2).

### 3.4. Moderation Effect of OE on the Association between the Perceived Efficacy of the COVID-19 Vaccination and Intention to Receive the COVID-19 Vaccination

**Figure 3:**
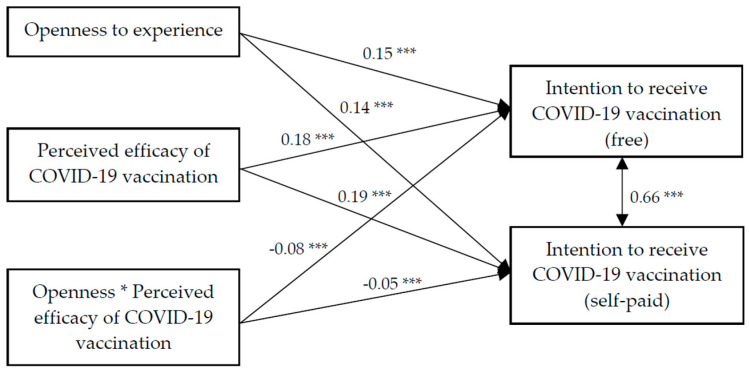
Path analysis on the moderation effect of openness experience on the association between the perceived efficacy of the COVID-19 vaccination and intention to receive the COVID-19 vaccination (free and self-paid). * *p* < 0.05, *** *p* < 0.001.

**Figure 4:**
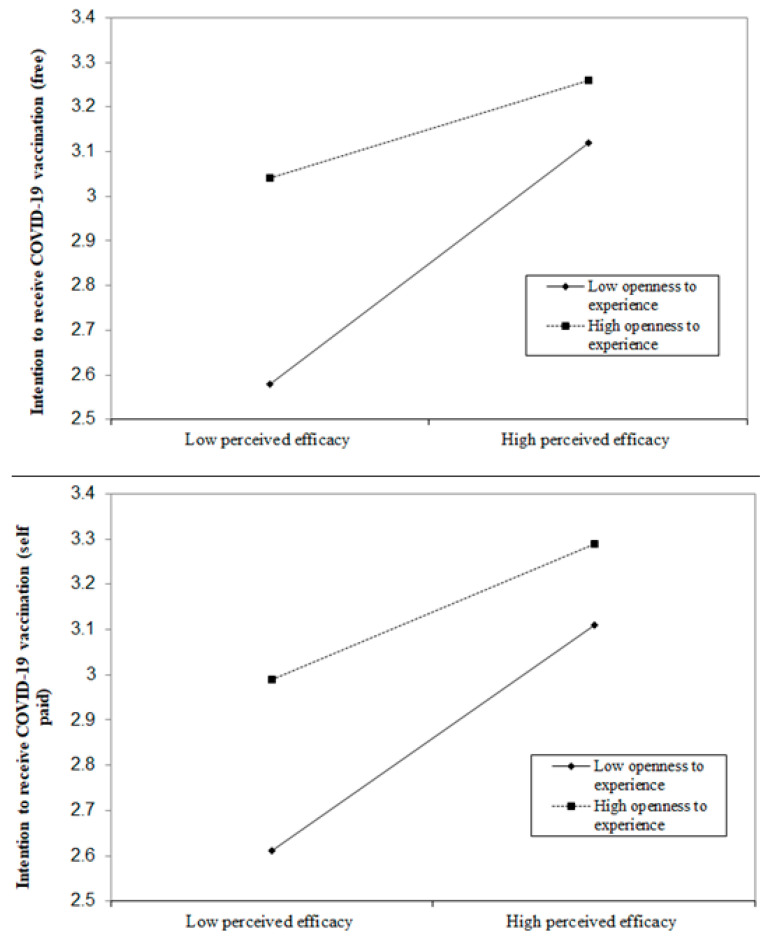
Moderating role of openness to experience on the association between the perceived efficacy of the COVID-19 vaccination and intention to receive the COVID-19 vaccination (free and self-paid).

Findings of the path analysis showed that the model had a satisfactory fit, χ^2^ (28) = 308.10, CFI = 0.98, IFI = 0.98, RMSEA = 0.04. OE, perceived efficacy, and the interaction term of OE and perceived efficacy of the COVID-19 vaccination all had significant association with the intention to receive the COVID-19 vaccination (free) (β = 0.15, 0.18, −0.08, *p* < 0.001, respectively) and intention to receive the COVID-19 vaccination (self-paid) (β = 0.14, 0.19, −0.05, *p* < *0*.01, respectively) (Figure 3). Further analyses showed that the positive association between the perceived efficacy of the COVID-19 vaccination and intention to receive the COVID-19 vaccination (both free and self-paid) was stronger among those with a lower level of OE (Figure 4).

The association between background variables and intention to receive the COVID-19 vaccination (free and self-paid) were omitted for clarity.

Moderation effect of OE on the association between social media use for COVID-19 vaccine information and intention to receive the COVID-19 vaccination.

Findings of the path analysis showed that the model had a satisfactory fit, χ^2^ (28) = 305.10, CFI = 0.97, IFI = 0.97, RMSEA = 0.04. OE and social media use for COVID-19 vaccine information had significant association with the intention to receive the tCOVID-19 vaccination (free) (β = 0.15, 0.03 *p* < 0.001, respectively) and intention to receive the COVID-19 vaccination (self-paid) (β = 0.14, 0.07 *p* < 0.01, respectively). However, the interaction term of OE and social media was not significant (data not tabulated).

### 3.5. Moderation Effect of OE on the Association between Descriptive Norm and Intention to Receive the COVID-19 Vaccination

**Figure 5:**
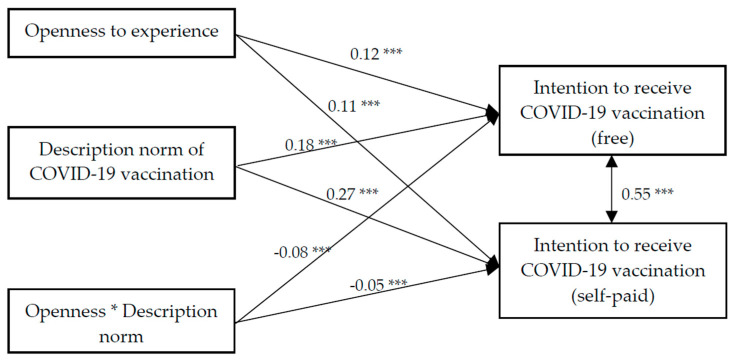
Path analysis on the moderation effect of openness experience on the association between descriptive norm and intention to receive the COVID-19 vaccination (free and self-paid). * *p* < 0.05, *** *p* < 0.001.

**Figure 6:**
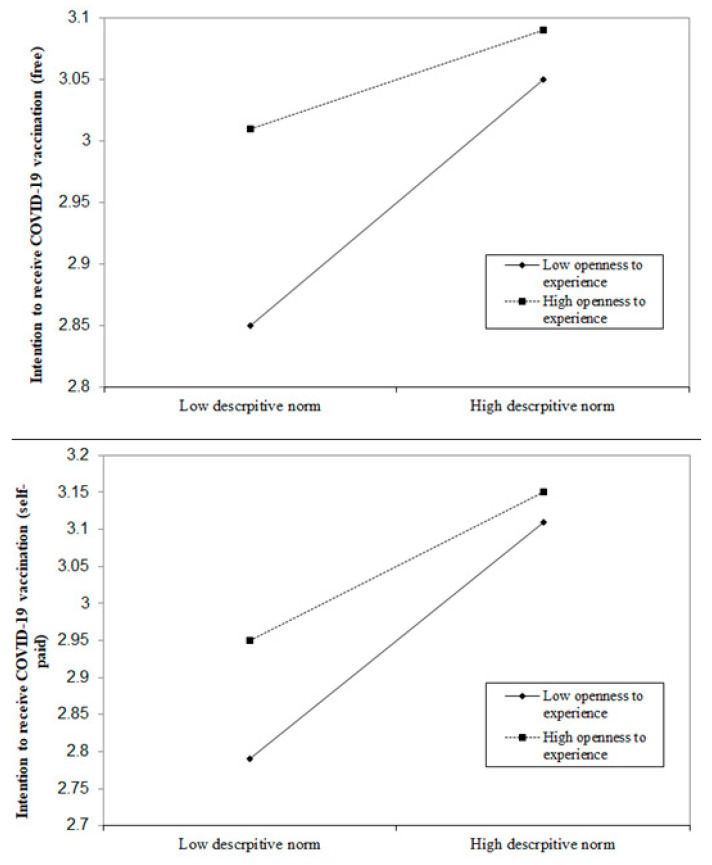
Moderating role of openness to experience on the association between descriptive norm and intention to receive the COVID-19 vaccination (free and self-paid).

Findings of the path analysis showed that the model had a satisfactory fit, χ^2^ (28) = 405.8, CFI = 0.97, IFI = 0.97, RMSEA = 0.04. OE, descriptive norm, and the interaction term of OE and descriptive norm all had significant association with the intention to receive the COVID-19 vaccination (free) (β = 0.12, 0.18, −0.08, *p* < 0.001, respectively) and intention to receive the COVID-19 vaccination (self-paid) (β = 0.11, 0.27, −0.05, *p* < 0.05, respectively) (Figure 5). Further analyses showed that the positive association between descriptive norm and intention to receive the COVID-19 vaccination (both free and self-paid) were stronger among those with a lower level of OE (Figure 6).

The association between background variables and intention to receive the COVID-19 vaccination (free and self-paid) were omitted for clarity.

## 4. Discussion

Vaccination is one of the most powerful public health interventions and an important means for preventing communicable infectious diseases. To maintain herd immunity, it is important to understand the factors that underlie public acceptability and adoption of novel vaccines. Previous studies have demonstrated the usefulness of DOI in understanding the adoption of innovative health behaviors. Using DOI as the theoretical framework, the present study examined the role of perceived efficacy of the COVID-19 vaccination (i.e., attribute of the innovation), use of social media for COVID-19 vaccine-related information (i.e., communication channel), OE (characteristics of the adopters) and descriptive norm (social system) on the intention to receive free and self-paid the COVID-19 vaccination among university students in China. Consistent with the extant literature that identified perceived benefits as critical factors affecting vaccination decisions [6,9,10,11,12,13,14,46], the present study showed that the perceived efficacy of the vaccine was positively associated with the intention to receive the COVID-19 vaccination. Despite its importance, skepticism and disbelief in the effectiveness and safety of the COVID-19 vaccination have also been documented in the literature [24,25]. Our findings accentuate the importance of increasing public education on vaccine efficacy and necessity as a means to reduce vaccine hesitancy and increase acceptance.

In an era when individuals are exposed to loads of information about the benefits and potential risk of vaccination over the Internet and social media, it is important to understand the degree to which exposure to social media for COVID-19 vaccine-related information will influence one’s beliefs and willingness to accept the COVID-19 vaccination. The present study revealed that using social media for COVID-19 vaccine-related information was associated with a higher intention to receive the vaccination. Our findings are in line with the literature that demonstrates the crucial role of social media in promoting healthly behavior [47,48]. Social media combines the advantages of mass media and interpersonal channels, and is believed to be particularly effective in creating awareness and knowledge, and forming and changing attitudes towards an innovation [19]. Individuals who accessed social media for COVID-19 related vaccination might have an increased understanding of the benefits and necessity of the COVID-19 vaccination, which promotes their willingness to receive the COVID-19 vaccination.

The present study also reported a positive association between descriptive norm and intention to receive the COVID-19 vaccination. Our findings provide the support that individuals’ perception of what other people will do is a powerful predictor of innovative behavior [40]. Since Chinese individuals tend to obey the authorities and conform to social norms [49,50], perceiving that others are also following the suggestion of receiving the COVID-19 vaccination might increase their likelihood to perform the behavior. Perceiving that more people in the social system are adopting an innovation also reduces the uncertainty associated with the novel ideas and increases someone’s belief that the behavior is appropriate, which subsequently promotes intention to engage in the behavior.

OE has been linked to the adoption of a range of innovation behaviors and positive health behaviors in the literature [34,35,36,37,38,39], its significant role in motivating intention to receive the COVID-19 vaccination has also been confirmed in the present study. Individuals who are high in openness tend to enjoy a variety of new experiences [51]. They may, therefore, be more likely to be receptive to innovative preventive strategies, such as the COVID-19 vaccination [52]. Furthermore, the present study also revealed a significant moderating role of OE, in which the association between the perceived efficacy of the tCOVID-19 vaccination and descriptive norm on the intention to receive the COVID-19 vaccination was stronger among those with lower OE. Our findings concur with previous findings that individuals who are open to new experiences are more likely to rely on personal inclination and emotions [33]. According to Roger, those who are more open to new ideas and experiences are more likely to understand and apply the skills essential for bridging in the innovation from outside their social system [19]. On the other hand, those who are less open tend to be less aware of the innovation, and thus, need to rely on the information of the innovation, and social norms to make their behavioral choice. Our findings provide an important indication that attitudes toward vaccines may be more easily changed based on scientific evidence and perception about norms among those who are low in OE.

### 4.1. Implications for Practice

With the rapid development of potential vaccines for COVID-19, there is an urgent need to understand the factors that predict COVID-19 vaccination intention, as such factors may guide the design of interventions to prepare for its public availability and maximize public acceptance [13]. The DOI provides a framework for the analysis of the promotion of innovations at a complex systems level, taking into account the differences in adopters, communication channels, and the specific attributes of an innovation that may affect diffusion. The findings of the present study provide empirical evidence and guidance for the design of health interventions based on the DOI. First, the findings of the present study demonstrated that perceived efficacy was an important attribute leading to acceptance of the COVID-19 vaccine. Our findings inform the need for health care providers and public health professionals to include discussions about the benefits of the vaccines and to clearly communicate the efficacy of it in preventing COVID-19 infection. Evidence has shown that providing the public information about the benefits and efficacy of a vaccine can effectively promote their intention to vaccinate. Public education about the efficacy of COVID-19 is of paramount importance.

The widespread use of social media has made it a ready platform for health promotion. The positive association between social media exposure to COVID-19 vaccine-related intention and willingness to receive vaccination has proven the promise to encourage the public to receive the COVID-19 vaccination through disseminating information about the COVID-19 vaccine on social media. Health care professionals should recognize the potential of social media for reaching a large group of audience and empowering them in their health care decision. It would be essential to take advantage of social media to publish professional advice and scientific knowledge about the COVID-19 vaccination. Message design should account for user characteristics, information preferences, mode of social media, and at the same time, ensure the information accuracy to improve communication effectiveness.

The significant role of descriptive norm evidenced in the present study suggests that more efforts should be placed on the influence of the social system when promoting the COVID-19 vaccination. There has been evidence that manipulating descriptive norms can effectively change a health behavior [53,54]. For example, one study among undergraduate students found that after seeing discarded wrappers indicating that other students typically had chosen a healthy or unhealthy snack as part of a taste test, students tended to select the snack that matched what they perceived to be the descriptive norm [54]. Drawing the public’s attention to the descriptive norm of the vaccination behavior is a relatively simple and inexpensive technique for modeling behavioral intention. Providing information that the majority of the people in the community are anticipated to receive the COVID-19 vaccination once it is available can normalize the vaccination behavior and potentially increase their intention.

Finally, findings also suggest that OE plays an important role in the diffusion process. Individuals who are open to new experiences are more likely to receive the COVID-19 vaccination, and their decision to be vaccinated is less likely to be affected by perceived norms and the attribute of the vaccination. Intervention that seeks to launch the new idea of the COVID-19 vaccine in the community should recruit individuals who are high in OE as they can serve as a gatekeeper and role model to introduce the new idea into their social system, and help to speed up the diffusion process. On the other hand, findings also reveal that individuals who are less open to new experiences tend to be more responsive to the descriptive norm and perceived efficacy of the vaccine in forming their vaccination intention. Interventions that promote the COVID-19 vaccination among people who are lower in OE should highlight the scientific evidence of the benefits of the vaccination, and the norm and subjective evaluation of the innovation by other community members.

### 4.2. Limitations

There are several limitations of the study. First, the study was cross-sectional in nature, so no causality between the variables can be assumed. Data was only obtained from five provinces, and our sample contained a relatively larger number of freshmen and students from the medical discipline, so it might not be representative of the university student population in mainland China. Participants were self-selected and self-reported measures were used; therefore, the intention to receive the COVID-19 vaccination and beliefs about the vaccine might have been over-estimated. Due to issues regarding the length of the questionnaire, a single item was used for most of the variables. The validity of such measures, therefore, should be cautioned. The present study included only university studies, who are young and more educated, and may be more likely to adopt an innovation (i.e., the COVID-19 vaccination). Future studies should include those with lower socioeconomic status, such as older populations, those living in rural areas, or individuals with a lower level of education and test the applicability of DOI in these populations. Furthermore, the present study was conducted among the Chinese population, who tends to be collectivistic and shows a higher tendency to respect the authorities and follow the social norms. Future studies should explore if the factors understudied could be applied to an individualistic culture. Lastly, future studies may also benefit from comparing participants’ intentions to receive COVID-19 vaccinations to the adoption of other innovations that have a less distinct effect on quality of life. Such comparison would provide more insights on the role of DOI in the adoption of innovations under different contexts.

## 5. Conclusions

The present study concluded that factors under the DOI, namely, perceived efficacy of the COVID-19 vaccination, social media use for COVID-19 vaccine-related information, OE, and descriptive norm, were all significant factors in influencing intention to receive the COVID-19 vaccination. The association between the perceptions of COVID-19 was also found to be stronger among those with a lower level of OE. Our findings underscore the importance of promoting the efficacy and norms of vaccination, and engaging people who are open to experience in the early diffusion process. Such health education effects can also take advantage of social media to promulgate the effects.

## Figures and Tables

**Table 1:** Demographic variables of the participants (*n* = 6922).

	*N* (%)
Gender	
Male	2520 (36.4%)
Female	4402 (63.6%)
Age (in years)	M = 19.4, SD = 1.51
Ethnicity	
Han	6009 (86.8%)
Others	913 (13.2%)
Discipline of study	
Arts	896 (12.9%)
Social Sciences	363 (5.2%)
Economics and management	378 (5.5%)
Science	703 (10.2%)
Engineering	819 (11.8%)
Medicine	3525 (50.9%)
Others	238 (3.4%)
Year of study	
First	2993 (43.2%)
Second	1894 (27.4%)
Third	1164 (16.8%)
Fourth	562 (8.1%)
Fifth	214 (3.1%)
Postgraduate	95 (1.3%)
Intention to receive the COVID-19 vaccination (score of 4 or above)	
Free vaccination	5464 (78.9%)
Self-paid vaccination	4165 (60.2%)

**Table 2:** Correlation between variables.

	1	2	3	4	5	6	7	8	9	10	11
1. Gender	-										
2. Age	0.003	-									
3. Ethnicity	0.031 *	0.050 ***	-								
4. Discipline of study	−0.045 ***	0.099 ***	−0.095 ***	-							
5. Year of study	0.026 *	0.806 ***	−0.014	−0.094 ***	-						
6. COVID-19 vaccination intention (free)	0.030 *	−0.023	−0.047 ***	0.036 **	−0.012	-					
7. COVID-19 vaccination intention (self-paid)	0.034 **	−0.058 ***	−0.004	0.008	−0.046 ***	0.581 ***	-				
8. Perceived efficacy of the COVID-19 vaccination	−0.057 ***	−0.012	−0.017	−0.002	0.000	0.228 ***	0.239 ***	-			
9. Social media use for COVID-19 vaccine-related information	−0.006	−0.019	0.032 **	−0.075 ***	−0.040 ***	0.046 ***	0.086 ***	0.109 ***	-		
10. OE	−0.025 *	−0.047 ***	−0.024 *	0.035 *	−0.041 ***	0.149 ***	0.158 ***	0.214 ***	0.104 ***	-	
11. Descriptive norm	−0.111 ***	−0.050 ***	0.033 **	−0.017 *	−0.063 ***	0.195 ***	0.281 ***	0.499 ***	0.138 ***	0.175 ***	-

* *p* < 0.05, ** *p* < 0.01, *** *p* < 0.001. Reference group: gender (male), ethnicity (Han), Discipline (Medicine).

## Data Availability

The data presented in this study are available on request from the corresponding author. The data not publicly available due to ethical consideration.
